# Canine distemper virus induces apoptosis in cervical tumor derived cell lines

**DOI:** 10.1186/1743-422X-8-334

**Published:** 2011-06-30

**Authors:** Helen L Del Puerto, Almir S Martins, Amy Milsted, Elaine M Souza-Fagundes, Gissandra F Braz, Barbara Hissa, Luciana O Andrade, Fabiana Alves, Daniela S Rajão, Rômulo C Leite, Anilton C Vasconcelos

**Affiliations:** 1 Department of General Pathology, Institute of Biological Science, Universidade Federal de Minas Gerais, UFMG, Brazil; 2 Department of Physiology, Institute of Biological Science, Universidade Federal de Minas Gerais, UFMG, Brazil; 3 Department of Biology, University of Akron, Akron, Ohio, USA; 4 Veterinary School, Department of Preventive Veterinary Medicine, Universidade Federal de Minas Gerais, UFMG, Brazil; 5 Department of Cellular Biology, Institute of Biological Science - Universidade Federal de Minas Gerais, UFMG, Brazil

**Keywords:** Apoptosis, Canine distemper virus, Caspase, Cervical tumor, HeLa cell, HPV

## Abstract

Apoptosis can be induced or inhibited by viral proteins, it can form part of the host defense against virus infection, or it can be a mechanism for viral spread to neighboring cells. Canine distemper virus (CDV) induces apoptotic cells in lymphoid tissues and in the cerebellum of dogs naturally infected. CDV also produces a cytopathologic effect, leading to apoptosis in Vero cells in tissue culture. We tested canine distemper virus, a member of the Paramyxoviridae family, for the ability to trigger apoptosis in HeLa cells, derived from cervical cancer cells resistant to apoptosis. To study the effect of CDV infection in HeLa cells, we examined apoptotic markers 24 h post infection (pi), by flow cytometry assay for DNA fragmentation, real-time PCR assay for caspase-3 and caspase-8 mRNA expression, and by caspase-3 and -8 immunocytochemistry. Flow cytometry showed that DNA fragmentation was induced in HeLa cells infected by CDV, and immunocytochemistry revealed a significant increase in the levels of the cleaved active form of caspase-3 protein, but did not show any difference in expression of caspase-8, indicating an intrinsic apoptotic pathway. Confirming this observation, expression of caspase-3 mRNA was higher in CDV infected HeLa cells than control cells; however, there was no statistically significant change in caspase-8 mRNA expression profile. Our data suggest that canine distemper virus induced apoptosis in HeLa cells, triggering apoptosis by the intrinsic pathway, with no participation of the initiator caspase -8 from the extrinsic pathway. In conclusion, the cellular stress caused by CDV infection of HeLa cells, leading to apoptosis, can be used as a tool in future research for cervical cancer treatment and control.

## Background

Apoptosis is a regulated form of cell death which occurs during physiological conditions. It plays a critical role in the homeostasis of multicelular organisms, and constitutes a common pathway for cell replacement, tissue remodeling, damaged cell removal and elimination of cancer cells [1]. It is a complex process which involves the participation of caspases, activation of proapoptotic genes, and inhibition of antiapoptotic proteins. Cells undergoing apoptosis present typical morphological characteristics, including membrane blebbing, chromatin condensation, cell shrinkage and apoptotic body formation [2].

Apoptosis is triggered by sequential activation of caspases, a group of cysteine proteases, and proceeds primarily through two pathways. The extrinsic pathway involves activation of caspase-8 and is initiated by ligand interaction with death receptors, while the intrinsic pathway is activated by an imbalance between proapoptotic and antiapoptotic proteins from Bcl-2 family in mitochondria and cytosol, resulting in release of cytochrome *c *from mitochondria, which in turn activates caspase-9 [3]. Both caspase-8 and caspase-9 activate caspase-3, which along with other effectors caspases cleave critical cellular proteins, resulting in apoptosis [3].

Many viral proteins can influence the cellular pathways that control cell proliferation and apoptosis. Some viral proteins trigger apoptotic cell death, and this may be important in host defense and viral spread. In other cases, viral proteins inhibit apoptosis [4].

HeLa cells, derived from a cervical tumor, encode apoptosis inhibitor proteins E6 and E7, oncoproteins expressed by high-risk human papillomavirus (HPV), among them the HPV18 type [5,6]. HPV E6 protein target p53, a tumor suppressor protein that regulates the cell cycle. The E6 protein binding to p53 causes p53 inactivation by its degradation, turning off its function [6,7]. On the other hand, the HPV E7 protein acts by binding to members of the Rb (retinoblastoma protein) tumor suppressor protein family, inhibiting its activity of controlling cell division [8-10]. HPV E6 and E7 are related to the resistence of HeLa cells apoptosis [11,12].

Canine distemper virus (CDV), a negative-stranded RNA belonging to the genus Morbillivirus, family Paramyxoviridae causes both cytopathologic infection and persistent infections in vivo and in vitro [13,14]. CDV has been shown to induce apoptosis in cerebellum and lymphoid tissue of naturally infected dogs [15], through the extrinsic pathway, activating caspase -8 and caspase -3 gene expression [16]. In addition, it was observed that CDV cause apoptosis of Vero cells, also by triggering the extrinsic pathway, with caspase -8 and caspase -3 activation [17,18].

The main objectives of this study were to determine if CDV is able to induce apoptosis in cultured HeLa cells, and use CDV and its proteins as a prospective research line for cervical cancer treatment and control.

## Materials and methods

### Cell Culture

HPV18-positive HeLa cells (ATCC^®^, CCL-2™, Virgínia, USA) were cultured on 24 well plates or in 33 and 100 mm plates (TPP^®^, Trasadingen, Switzerland) to approximately 70%-80% confluence in DMEM medium (Sigma) supplemented with 10 mmol/l HEPES and 5% fetal bovine serum (Atlanta Biologicals) in a humidified atmosphere at 37°C and 5% CO_2_.

### CDV infection of HeLa cells

Prior to CDV infection, approximately 25.000 HeLa cells/cm^2 ^were seeded into 24 well cassettes for immunocytochemistry assay, on 100 mm plate for mRNA analysis, and on 33 mm plate for flow-cytometry analysis and incubated overnight. Each well/plate, with approximately 70-80% of confluence, was infected with 300 μl of CDV-Lederle strain, in a proportion of 2 parts serum free medium to 1 part CDV, at a multiplicity of infection of 0.1. After 1 h of virus adsorption, media was removed and fresh complete medium was applied. After 24 h the cells were processed for immunocytochemistry, flow-cytometry and real-time PCR procedures.

### Immunocytochemistry for caspase -3 and -8

After HeLa infection medium was removed at 24 hours post infection, and cells washed 2 X with phosphate buffered saline (PBS) containing 0.9 mM CaCl_2 _and 0.5 mM MgCl_2_. Cells fixed with 4% paraformaldehyde for at least 4 hours at 4°C. Coverslips containing fixed cells were washed three times with Tris buffered saline (TBS-50 mM Tris-HCl, 0,15 M NaCl, 2%) pH 7.6, and then permeabilized with TBS containing 2% Bovine serum albumin (BSA) and 0.1% Triton X for 10 minutes. The first step was the inhibition of endogenous peroxidase in order to avoid its reaction with the substrate outside of the specific antigenic sites. For this, cells were incubated for 5 minutes with Peroxidase Block (NovocastraTM Peroxidase Detection System - RE7101), and then washed twice with TBS. The second step was the incubation for 5 minutes with a blocking protein (NovocastraTM Peroxidase Detection System - Protein Block - RE7102) to suppress non-specific binding of subsequent reagents. Subsequently cells were washed twice with TBS for 5 minutes.

For caspase-3 localization, a monoclonal mouse anti-human antibody (Novocastra Laboratories, Newcastle, UK) was diluted 1:50 and applied for 1 hour at room temperature in a moist chamber. This antibody recognizes caspase-3 protein in its active form. For caspase-8 localization, a monoclonal mouse anti-human caspase-8 antibody (Novocastra Laboratories, Newcastle, UK) was diluted 1:30 and applied for 1 hour at room temperature in a moist chamber. This antibody recognizes caspase-8 protein in its active form. Cells were washed with TBS, and incubated with biotinylated secondary antibody (NovocastraTM Peroxidase Detection System - RE7103), for 30 minutes at room temperature. After incubation and washing the excess secondary antibody with TBS, cells were then incubated for 30 minutes with streptavidin-HRP (NovocastraTM Peroxidase DetectionSystem-RE7104). Finally, after washing cells with TBS, cells were stained with 3,3 '-diaminobenzidine (DAB Chromogen) (NovocastraTM Peroxidase Detection System) in a buffer solution (NovocastraTM Peroxidase Detection System - RE7106).

The negative control consisted of HeLa cells incubated with TBS+2%BSA, and positive control consisted of HeLa cells incubated with 50 μM cisplatin. Caspase-3 positive cells were identified by the presence of a brown color staining with sharp outlines and homogeneous in the nucleus and cytoplasm; caspase-8 positive cells were identified by the presence of a brown color staining with sharp outlines and homogeneous in cytoplasm.

### DNA fragmentation assay - Flow-Cytometry

Cell cycle status and quantification of DNA fragmentation (subdiploid DNA-content), apoptosis morphological and biochemical characteristic, were performed by propidium iodide (PI) staining according to Nicolleti et al, 1991 [19]. After 24 hours of CDV infection, media was removed, and floating and adherent cells were lysed with 200 μl of a hypotonic fluorochrome solution - HFS (50 μg/mL PI in 0.1% sodium citrate plus 0.1% Triton X-100) added onto a HeLa cell monolayer, protected against light, and incubated at 4°C for 4 h. HeLa cells in the supernatant of the removed media were centrifuged, 100 μl of HFS solution added, and cells were incubated at 4°C for 4 h. The PI fluorescence of 20,000 individual nuclei was measured using a FACScalibur flow cytometer (Becton Dickinson Immunocytometry Systems, California, USA). Data were analyzed using FlowJo software (TreeStar Inc, CA). For the positive control for apopotosis, HeLa cells treated with 40 μM of cisplatin were used. Cisplatin is an antitumor drug known for inducing apoptosis in cancer cell line [20]. For negative control HeLa cells with no treatment were used. All samples were run in triplicate in at least three independent experiments.

### Real time PCR for caspase-3 and -8

#### Isolation of RNA

After 24 hours of CDV infection, HeLa cells were washed with sterile PBS. Total RNA was isolated adding 1 ml of Trizol^® ^reagent (Invitrogen Life Technologies, Carlsbad, CA, United States of America - USA) to each 100 mm dish, following Trizol^® ^manufacturer's protocol. At the end, the RNA pellet was briefly dried, redissolved in RNase and DNase free water (Invitrogen Life Technologies, Carlsbad, CA, United States of America - USA), and quantified using NanoDrop (Thermo Fisher Scientific, Wilmington, Delaware, USA). RNA samples were treated with Turbo DNA-free kit (Ambion Inc., Foster, CA, USA), and stored at -80°C until use.

#### Reverse transcription (RT)

First-strand complementary DNA (cDNA) was synthesized from 2 μg total RNA using the Superscript first-strand synthesis system (Invitrogen Inc., Carlsbad, CA, USA). After denaturing the RNA template and primers (25 pmol of each reverse oligonucleotide primer) were incubated at 70°C for 10 min, and 40 U reverse transcriptase was added in the presence of RT buffer (50 mM KCl, 20 mM Tris-HCl, pH 8.4), 4 μL dNTP mix (250 μM each), 40 U RNase inhibitor and RNase-free water to complete the final volume. The reaction mixture (50 μL) was incubated at 43°C for 1 h, then stopped at 4°C and used for PCR.

#### Real time PCR

Real-time PCR was carried out in an ABI Prism 7500 Sequence Detection System (Applied Biosystems, Foster City, CA, USA), using the *Power *Sybr^® ^Green Master Mix Kit (*Invitrogen Life Technologies*, Carlsbad, CA, USA).. All samples were run in duplicate. The PCR parameters were 1 cycle at 50°C for 2 min, 1 cycle at 95°C for 10 min, 40 cycles at 95°C for 15 s and 60°C for 1 min. Primers used for PCR amplification of caspase-3, -8 and Gapdh, are listed on Table 1. Gapdh (Glyceraldehyde-3-Phosphate Dehydrogenase) was used as a reference transcript to normalize target transcript expression.

**Table 1:** Sequences of primers used in real time PCR

Primers	Nucleotide Sequence	Length (nt)	Fragment size	GenBank access number
**HsCasp3Forw**	5'- TGCATACTCCACAGCACCTGGTTA-3'	24	82 bp	NM_032991.2
**HsCasp3Rev**	5'-CATGGCACAAAGCGACTGGATGAA-3'	24		

**HsCasp8Forw**	5'- TTTCACTGTGTTAGCCAGGGTGGT A-3'	24	84 bp	NM_033355.3
**HsCasp8Rev**	5'- CCTGTAATCCCAGCACTTTGGGAG -3'	24		

**HsGapdhForw**	5'- TTCCAGGACCAAGATCCCTCCAAA - 3'	24	86 bp	XM_001725661
**HsGapdhRev**	5'- ATGGTGGTGAAGACACCAGTGAAC - 3'	24		

### Statistical analysis

DNA fragmentation assay results were expressed as the mean ± SD of three independent experiments carried out in triplicate. These data were analyzed using Student's t-test for paired comparisons between infected cells and controls. Statistical significance was determined as p < 0.05.

PCR results were analyzed based on the ΔCT, which is the primary source of data variability [21]. The CT values were normally distributed and therefore they were summarized as mean ± standard error and differences between the two groups were analyzed with the unpaired Student's t test, considering p < 0.05 as statistically significant [21].

## Results

### Immunocytochemistry

**Figure 1:**
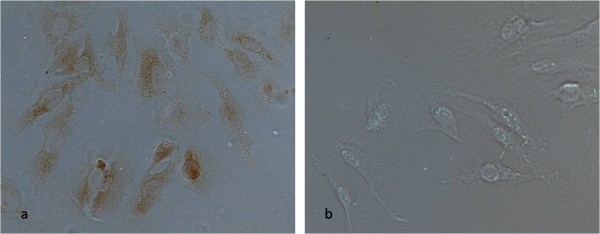
**Immunocytochemistry of CDV infected cells**: Immunolocalization of caspase-3 (a) and caspase-8 (b) in HeLa cells infected by CDV. The nuclear and cytoplasmatic brownish staining indicates occurrence of active caspase-3 in infected cells (a). No active caspase-8 is observed for infected cells (b) (Microscope objective 40 × 0.75).

**Figure 2:**
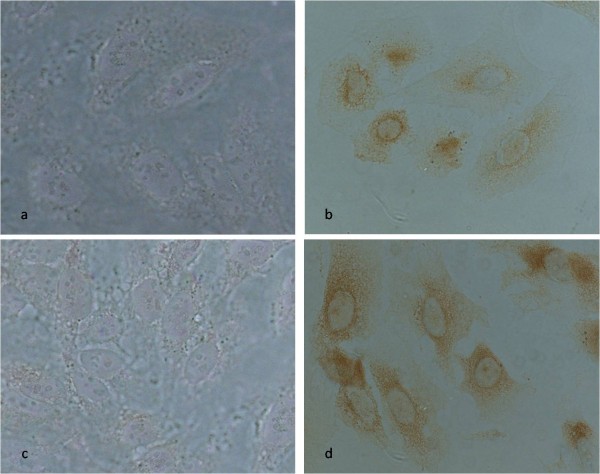
**Immunocytochemistry positive and negative control**: Immunolocalization of caspase-3 and caspase-8 (b *and *d) in HeLa cells treated with 40 μM of cisplatin. Nuclear and cytoplasmatic brownish staining is observed for treated cultures indicating occurrence of active caspase-3 (b) and caspase-8 (d) (Microscope objective 40 × 0.75). No staining for active caspase-3 (a) or caspase-8 (c) was observed for control non-treated cells (Microscope objective 63 × 1.23).

**Figure 3:**
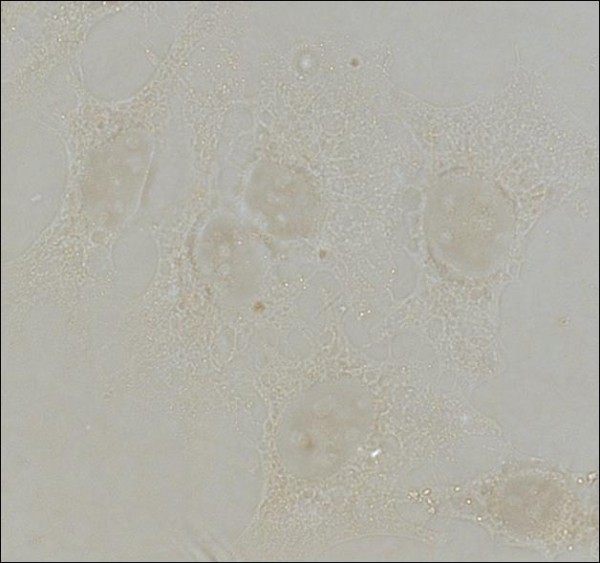
**Immunocytochemistry experiment control**: No staining was observed in the experiment negative control consisted of HeLa cells treated with cisplatin (40 μM) incubated with TBS+2%BSA, instead the primary antibody for caspase -3 or -8 (Microscope objective 63 × 1.23).

Nuclear and cytoplasmatic immunoreactivity for caspase-3 was present in HeLa cells infected by CDV, as well as in control group treated with cisplatin (Figure 1a and 2b). There was no immunoreactivity for caspase-8 in infected HeLa cells (Figure 1b); however it was shown in HeLa cells treated with cisplatin (Figure 2d). Control HeLa cells with no treatment were both, caspase -3 and -8 negative (Figure 2a and 2c). Reaction control of HeLa cells treated with 40 μM of cisplatin incubated with TBS+2%BSA, instead the primary antibody, confirmed that there was no nonspecific staining of the cells by the secondary antibody (Figure 3).

### DNA fragmentation assay - Flow-Cytometry

**Figure 4:**
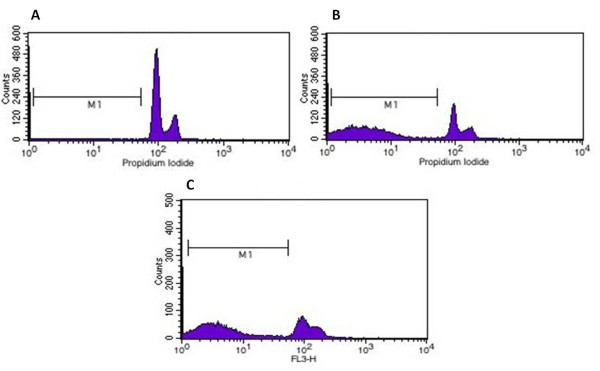
**Flow-Cytometry histogram**: Subdiploid DNA content was assessed by staining with PI and flow cytometry analysis. CDV infection induces apoptosis in HeLa cells (B). Cisplatin, positive control is demonstrated to cause apoptosis in positive control group (C). Negative control did not show subdiploid DNA content (A). Subdiploid DNA content indicates DNA fragmentation, typical in apoptotic cells.

To investigate whether apoptosis was triggered by CDV infection, DNA fragmentation and chromatin condensation were evaluated at 24 hours p.i. Significant increases in DNA fragmentation were detected in HeLa cells infected with CDV (57.68%) when comparing non-infected cells (6.97%) (Table 2) (P < 0.05, Student's t-test). Subdiploid DNA content indicating DNA fragmentation, typical in apoptotic cells, was observed in HeLa cells infected by CDV and treated with cisplatin (Figure 4).

**Table 2:** DNA fragmentation in HeLa cells infected by CDV.

Treatment	HeLa
Control	6.97 ± 3.56
Cisplatin	66.74 ± 3.32*
CDV	57.68 ± 3.08*

HeLa cells were incubated with 40 μM of cisplatin, CDV and vehicle (control), at 37°C in a 5% CO2 atmosphere for 24 hours. DNA content was assessed by flow-cytometric analysis of cells labeled with propidium iodide. Each data represents the mean ± SD from 3 different experiments (* P < 0.05, Student T-test).

### Real time PCR for caspase-3 and -8

**Figure 5:**
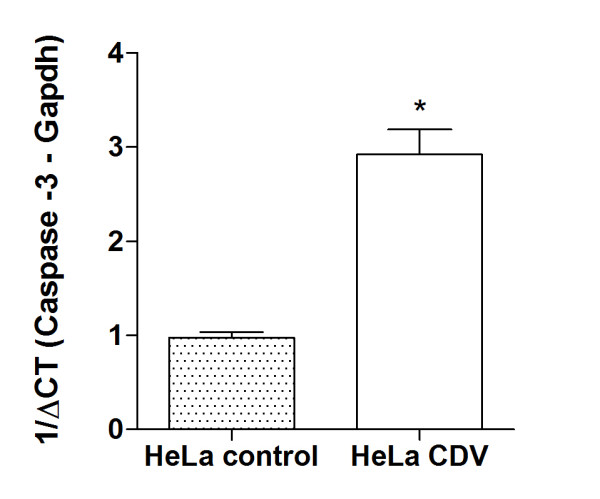
**Real time PCR results**: Expression of caspase-3 mRNA in HeLa cells infected by CDV. For better clarity, data are plotted as mean ± standard error of 1/ΔCT, which is directly proportional to the relative gene expression (fold change). *p < 0.05 (paired t test - GraphPad Prism5.0).

Real-time PCR was performed to measure mRNA expression using specific primers for caspase-3, caspase-8 and the reference gene Gapdh. Expression of caspase-3 mRNA increased in the CDV infected HeLa cell group when compared to control cells (Figure 5). Interestingly, caspase-8 mRNA expression did not change in CDV infected HeLa cells compared to control cells.

## Discussion

In summary, our results show that CDV infection in HeLa cells induced apoptosis, probably by the intrinsic pathway, ending with the activation of the effectors' caspase -3, as was observed in immunocytochemistry, and real time PCR results.

HeLa cells, derived from a cervical tumor, encode apoptosis inhibitor proteins E6 and E7, oncoproteins expressed by high-risk HPV, among them the HPV18 type [5,6]. HPV E6 protein target p53 is a tumor suppressor protein that regulates the cell cycle. The E6 protein binding to p53 causes p53 inactivation by its degradation [6,7]. On the other hand, the HPV E7 protein acts by binding to members of the Rb tumor suppressor protein family, which contribute to the control of the cell division, process by modulating the function of E2F transcription factors [8-10].

Apoptosis plays important roles in host defenses against virus infection. Virus infected cells can show changes in physiology. Cells may recognize virus particles at cell entry and viral proteins during viral replication and, in response, execute apoptosis in order to block virus replication [22]. In addition, viral proteins can interact with cell components which regulates cell death, affecting apoptosis [23]. On the one hand it seems that virus proteins block apoptosis to prevent death of the host cell and so maximize virus replication and facilitate a persistent infection. On the other hand it seems that a growing number of viruses actively promote culminating a lytic infection and serving to spread virus to neighbouring cells while evading host infammatory responses [23-25].

It have been shown that apoptosis induced by canine distemper virus occurs by the extrinsic pathway, activating initiator caspase-8 followed by the activation of executioner caspase-3 [16,18]. Conversely, apoptosis in HeLa cells caused by CDV infection did not show the participation of caspase-8. From this, we can infer that the CDV mechanisms of apoptosis in HeLa cells are different from the apoptosis mechanisms triggered *in vivo *and in Vero cell line. However, HeLa CDV infection may cause intracellular stress, which culminates with the activation of the caspase cascade, leading to activation of the executioner caspase-3, and consequently cell death. So, even the presence of oncoproteins E6 and E7, which acts inactivating p53 and Rb proteins, did not inhibit the activation of caspases [5,8]. Caspases are initiators and effectors arm proteins of apoptosis, and once the caspase cascade is activated, the process of cell death is irreversible [3,26,27]. In conclusion, canine distemper virus infection seems to activate the effector caspase-3, leading to HeLa cells apoptosis, even in the presence of E6 and E7 oncoproteins.

## Conclusions

A relationship between viral infection and apoptosis has been reported, with some viral infections inducing apoptosis, while other inhibits apoptosis [7,15,24,25,28,29]. Several viral proteins can induce apoptosis of infected cells, such as VP3 protein of chicken anemia virus (CAV), EIA protein of the adenovirus, HA protein of influenza virus [28,30,31], and HIV [32]. There is no previous report identifying which CDV protein could be involved in the apoptosis induction *in vivo*, and *in vitro*. Further study is needed to evaluate the CDV proteins, to specify which protein could be involved and how it is related to apoptosis. Based on the present findings we concluded that CDV infection induces apoptosis in HeLa cells, by the intrinsic pathway, through the activation of executor caspase-3, without the participation of the initiator caspase-8.

## Competing interests

The authors declare that they have no competing interests.

## Authors' contributions

HLDP, ASM, ACV, AM and RCL designed and directed studies, and were involved in interpretation of the data and writing of manuscript. HLDP, FA and DSR developed primers and PCR procedures and performed the analysis of the PCR data. HLDP and EMSF performed the flow-cytometry experiments. HLDP, BH, GFB and LOA performed cell cultures, CDV infection and immunocytochemistry experiments and ran the assays. All authors have read and approved manuscript.
